# A predictive model of pregnancy loss using pre-pregnancy endocrine and immunological parameters in women with abnormal glucose/lipid metabolism and previous pregnancy loss

**DOI:** 10.1007/s12020-024-03937-7

**Published:** 2024-06-19

**Authors:** Fangxiang Mu, Mei Wang, Xianghui Zeng, Lin Liu, Fang Wang

**Affiliations:** Department of Reproductive Medicine, Lanzhou University Second Hospital, Lanzhou, 730030 China

**Keywords:** Abnormal glucose/lipid metabolism, Previous pregnancy loss, Predictive model, Nomogram, Pre-pregnancy endocrine parameters

## Abstract

**Objective:**

To investigate the clinical and endocrine risk factors for pregnancy loss in women with abnormal glucose/lipid metabolism and a history of pregnancy loss, and to develop a predictive model to assess the risk of pregnancy loss in these women’s subsequent pregnancies.

**Methods:**

Patients with a history of pregnancy loss who had abnormal glucose/lipid metabolism were retrospectively included in this study, and their pre-pregnancy baseline and clinical characteristics were collected. A predictive nomogram was constructed based on the results of the multivariable logistic regression model analysis, and its calibration and discriminatory capabilities were evaluated. The internal validation was then performed and the net benefits were assessed by the clinical decision curve.

**Results:**

The predictive model was eventually incorporated eight variables, including maternal age, previous pregnancy losses, anticardiolipin antibody (aCL) IgG, aCL IgM, thyroid peroxidase antibody, complement 4, free thyroxine and total cholesterol. The area under the curve (AUC) of the nomogram was 0.709, and Chi-square value and *P* value of the Hosmer–Lemeshow test were 12.786 and 0.119, respectively, indicating that the nomogram had a satisfactory calibration and discriminatory performance. The validation cohort showed a similar result for the discrimination of the nomogram (AUC = 0.715). The clinical decision curve demonstrated the nomogram had good positive net benefits.

**Conclusions:**

This is the first study to predict the risks of subsequent pregnancy loss in women with abnormal glucose/lipid metabolism and history of pregnancy loss using pre-pregnancy clinical and endocrine parameters. This predictive nomogram may provide clinicians assistance to personalize the management of subsequent pregnancies in these patients.

## Introduction

Pregnancy loss is a common obstetric complication, with an ~10% risk of one pregnancy loss in women of childbearing age [1]. Meanwhile, recurrent pregnancy loss (RPL), defined as two or more pregnancy losses before 24th week of gestation, occurs in 1~5% of couples, and its risk increases with the number of previous pregnancy losses [2, 3]. The absence of timely interventions may end with some severe outcomes, which can be a financial, physical, and emotional burden for the affected family. Immunological factors, thrombophilia, and chromosomal abnormalities are all common causes of pregnancy loss. Many factors associated with RPL, such as antiphospholipid antibodies (aPLs), are also linked to late pregnancy loss. Patients with positive aPLs have a significantly increased risk of adverse pregnancy outcomes [4].

Endocrine system diseases are also important factors associated with adverse pregnancy outcomes, such as thyroid dysfunction and glucose/lipid metabolism disorders. Thyroid hormone disorders and thyroid peroxidase autoantibodies (TPO-Ab) in women are associated with low fertility and pregnancy loss [5]. Studies showed that the history of pregnancy loss may also increase the risk of developing gestational diabetes mellitus during subsequent pregnancy [6]. Uncontrolled diabetes mellitus is relevant with a 50% chance of pregnancy loss in pregnant women [7]. Moreover, hyperinsulinemia may induce the occurrence of pregnancy loss by impairing ovum quality and endometrial tolerance, alongside elevated insulin can also cause elevated plasma total glyceride (TG) and total cholesterol (TC) levels and decreased high-density lipoprotein levels. Besides, obese women are more likely to suffer complications including hypertension, diabetes mellitus, and hypothyroidism, all of which further increase the risk of pregnancy, as in the case of postpartum hemorrhage and neonatal death [8, 9]. Therefore, preconception counseling for women with previous pregnancy loss and abnormal glucose/lipid metabolism would support their further understanding of pregnancy and effective pregnancy management. Few studies have examined the relationship between pre-pregnancy levels of endocrine and immunological factors and the occurrence of subsequent pregnancy loss in patients with a history of pregnancy loss, as most studies have investigated the risk of pregnancy loss associated with these factors during pregnancy. So, if the preconception test results of these potential risk factors can be used to predict pregnancy outcomes, and targeted treatment (if necessary) can be given before pregnancy, it would be beneficial to optimize the maternal-fetal outcomes.

Nomograms can accurately assess risks in an individualized manner and can be used to guide patient management and related decision-making. Specifically, it is an ideal prediction tool in clinical practice. Therefore, we aimed to investigate the factors influencing subsequent pregnancy loss in patients with a history of previous pregnancy loss and abnormal glucose/lipid metabolism during pregnancy and to develop a predictive model for individualized assessment of subsequent pregnancy loss risk.

## Methods

### Patient population

Patients with a history of pregnancy loss were retrospectively recruited from the Reproductive Medicine Center, the Lanzhou University Second Hospital (Gansu, China) between February 2019 and December 2022. Among them, 1955 patients (February 2019 to February 2022) were assigned into the training cohort, and 535 patients (February 2022 to December 2022) were assigned into the internal validation cohort. We completed the follow-up of all these 2490 patients in June 2023. The study was approved by the Ethics Committee of Lanzhou University Second Hospital (2019A-231).

Patients were diagnosed with abnormal glucose/lipid metabolism in accordance with the Guideline for the Prevention and Treatment of Type 2 Diabetes Mellitus in China (2020 edition) and Chinese Guidelines for Lipid Management (2023 edition). The included patients had glucose metabolism disorders, lipid metabolism disorders, or obesity (body mass index [BMI] ≥28 kg/m^2^ [10, 11]). Patients aged over 18 years and had a history of one or more pregnancy losses were eligible for this study. We excluded the patients with any of the following: (1) abnormal chromosome karyotype in a couple and/or pregnancy products; (2) medically documented history of antiphospholipid syndrome (APS) and other autoimmune diseases; (3) congenital uterine malformations (septate uterus, unicornuate uterus, bicornuate uterus and duplex uterus); and (4) polycystic ovary syndrome (PCOS) [12]. Additionally, women who had adverse pregnancy outcomes such as hydatidiform mole, biochemical pregnancy, and ectopic pregnancy were excluded. Moreover, women with no fertility plan, infertility, lost to follow-up, and incomplete medical records were not analyzed in this study.

### Definitions

In the present study, pregnancy loss was defined as the spontaneous demise of a pregnancy before 24 weeks of gestation according to the European Society of Human Reproduction and Embryology (ESHRE) guideline [3]. Glucose metabolism disorders included the status of impaired fasting glucose (6.1 ≤ FBG [fasting blood glucose] < 7.0 mmol/L and 2hPG [2-hours plasma glucose in the 75 g oral glucose tolerance test] <7.8 mmol/L), impaired glucose regulation (FBG < 7.0 mmol/L and 7.8 ≤ 2hPG < 11.1 mmol/L), diabetes mellitus (FBG ≥ 7.0 mmol/L and 2hPG ≥ 11.1 mmol/L), and other abnormal glucometabolic disorders [13]. Lipid metabolism disorders contained the following conditions: hypercholesterolemia (TC ≥ 5.2 mmol/L), hypertriglyceridemia (TG ≥ 1.7 mmol/L), combined hyperlipidemia (TC ≥ 5.2 mmol/L and TG ≥ 1.7 mmol/L), and low high-density lipoprotein cholesterol (HDL-C) (≤1.0 mmol/L) [14].

### Candidate predictors

Overall, the current study collected the following potential risk factors of pregnancy outcomes, including demographic characteristics (maternal age, BMI, education levels, regular menstruation, dysmenorrhea degree), previous live births and pregnancy losses, blood glucose indicators (FBG, 2hPG), blood lipid indicators (TC, TG, low-density lipoprotein cholesterol, HDL-C), thyroid function test (thyroid-stimulating hormone, free thyroxine [FT-4], free triiodothyronine, thyroglobulin antibodies, TPO-Ab), coagulation function tests (prothrombin time [PT], activated partial thromboplastin time, fibrinogen, thrombin time, D-dimer, fibrinogen degradation products), immune parameters (antinuclear antibodies, IgA, IgG, IgM, complement 3 (C3), complement 4 (C4), anticardiolipin antibody (aCL) IgG, aCL IgM, anti-β2 glycoprotein 1 antibody IgG, lupus anticoagulant screening/confirmatory test), and homocysteine and 25-hydroxyvitamin D levels.

### Statistical analysis

All statistical analyses were performed using R Studio (version 2022.02.1 + 461) and R (version 4.2.3; R Foundation for Statistical Computing, Vienna, Austria). Group *t-*test, Wilcoxon rank sum test, Chi-square test, and Fisher’s exact test were employed to compare the baseline characteristics and clinical outcomes. Data were given as mean ± standard deviation or frequency with percentages. *P* < 0.05 was determined as statistical significance.

### Model development

The primary outcome for this study was the pregnancy loss rates of patients with previous pregnancy loss and abnormal glucose/lipid metabolism. The predictive model was established based on the data from the patients who finally included in the study. We first applied the “stepAIC” function from “rms” package to screen the variables—with the highest predictive value as predictors of pregnancy loss—from the included patient characteristics by a backward multinomial logistic regression. The correlation between the variables was determined using the “corrplot.mixed” function of R. Then, the variables with a *P* < 0.05 in univariable analyses were selected. The predictors incorporated in the final nomogram included the results of multinomial logistic regression and univariable analyses. A final nomogram was created to visually represent the predictive model using the “regplot” function of R.

### Model evaluation

Model performance was assessed by the discrimination and calibration analyses. The accuracy of this predictive model was determined by measuring the area under the receiver operator characteristic (ROC) curve. Calibration was evaluated via the Chi-square value and *P* value of the Hosmer–Lemeshow test statistics. The internal validation of the training queue employs a tenfold cross-validation repeated 50 times, and the results of accuracy and kappa value was employed to evaluate the model performance. We further conducted an external validation for the developed predictive model using the validation cohort. Besides, a clinical decision curve was constructed to reflect the net benefits of patients at various risk thresholds, which means the number of correctly identified patients per hundred. The ROC curve was generated utilizing the “roc” function from the “pROC” package; calibration curve was plotted by the “calibrate” function from the “rms” package, with an upper limit of 1000 resampling; and clinical decision curve was created via the “decision_curve” function from the “rmda” package.

## Results

**Fig. 1:**
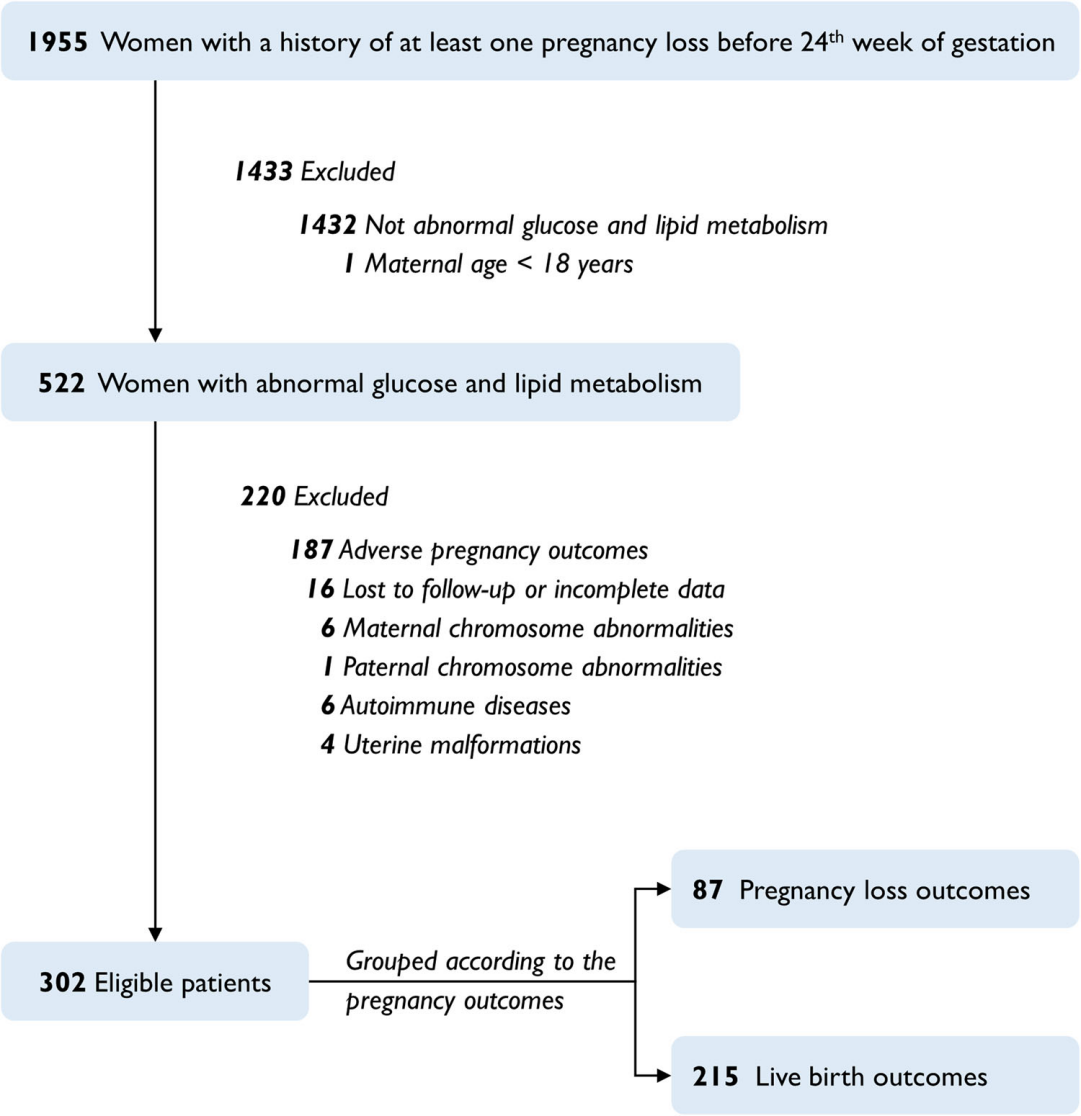
Flow chart for patient selection in the training cohort

Overall, 302 patients of training cohort fulfilled the inclusion criteria were eventually recruited in the study, and their pregnancy outcomes were 215 live births and 87 pregnancy losses (detailed in Fig. 1). Seventy-seven patients in the validation cohort were finally included, after excluding 395 patients who did not meet the criteria for abnormal glucose/lipid metabolism defined in this study, 2 with chromosome abnormalities, 3 with ectopic pregnancy, 3 with PCOS, and 73 who were lost to follow-up.

### Patients’ characteristics

Table 1 shows the patients’ baseline characteristics and clinical outcomes in the patients of training cohort and validation cohort. As shown, the age of menarche (13.43 ± 1.34 vs. 12.25 ± 3.77 years), regular menstruation (85.4% vs. 75.3%), previous live births, positive aCL IgG (26.5% vs. 15.6%), PT (11.11 ± 0.96 vs. 12.56 ± 11.46 s), and levels of IgA (12.64 ± 2.51 vs. 10.22 ± 4.51 g/L), IgG (2.27 ± 0.8 vs. 3.94 ± 3.81 g/L), C3 (1.28 ± 0.22 vs. 1.39 ± 0.21 g/L), TC (4.15 ± 0.98 vs. 4.4 ± 0.95 mmol/L), HDL-C (1.22 ± 0.32 vs. 1.51 ± 0.66 mmol/L) and 25-hydroxyvitamin D levels (12.47 ± 6.13 vs. 15.95 ± 7.46 ng/mL) were significantly different between the two cohorts (all *P* < 0.05).

**Table 1:** Comparison of baseline and clinical characteristics of training cohort and validation cohort

Variables	Training cohort N = 302	Validation cohort N = 77	*P* value
Maternal age (years)	30.89 ± 4.31	31.79 ± 4.25	0.100
BMI (kg/m^2^)	24.43 ± 3.67	24.54 ± 3.65	0.829
Age of menarche (years)	13.43 ± 1.34	12.25 ± 3.77	<0.001
Regular menstruation	258 (85.4)	58 (75.3)	0.033
Dysmenorrhea degree			0.305
Never	115 (38.1)	21 (27.3)	
Slight	139 (46.0)	44 (57.1)	
Obvious	31 (10.3)	8 (10.4)	
Serious	17 (5.6)	4 (5.2)	
Education level			0.276
Primary school	8 (2.6)	3 (3.9)	
Middle school	39 (12.9)	16 (20.8)	
High school	52 (17.2)	8 (10.4)	
Undergraduate	193 (63.9)	48 (62.3)	
Postgraduate	10 (3.3)	2 (2.6)	
Previous live births			0.006
0	262 (86.8)	57 (74.0)	
1	37 (12.3)	16 (20.8)	
2	2 (0.7)	4 (5.2)	
3	1 (0.3)	0 (0)	
Previous pregnancy losses			0.495
1	125 (41.4)	34 (44.2)	
2	117 (38.7)	29 (37.7)	
3	39 (12.9)	9 (11.7)	
≥4	21 (7.0)	5 (6.5)	
Type of previous pregnancy losses			0.051
Primary	259 (85.8)	59 (76.6)	
Secondary	43 (14.2)	18 (23.4)	
FBG (mmol/L)	5.18 ± 0.97	5.11 ± 1.53	0.616
2hPG (mmol/L)	7.18 ± 2.6	7.7 ± 3.43	0.141
TC (mmol/L)	4.15 ± 0.98	4.4 ± 0.95	0.046
TG (mmol/L)	1.74 ± 1.32	1.67 ± 0.92	0.698
HDL-C (mmol/L)	1.22 ± 0.32	1.51 ± 0.66	<0.001
LDL-C (mmol/L)	2.73 ± 0.79	2.59 ± 0.77	0.167
TSH (μIU/mL)	2.6 ± 1.44	2.54 ± 1.93	0.796
FT-3 (pmol/L)	5.27 ± 1.18	5.73 ± 5.71	0.187
FT-4 (pmol/L)	16.24 ± 4.05	16.55 ± 2.74	0.527
TG-Ab (+)	58 (19.2)	16 (20.8)	0.756
TPO-Ab (+)	61 (20.2)	20 (26.0)	0.270
PT (s)	11.11 ± 0.96	12.56 ± 11.46	0.029
APTT (s)	29.84 ± 5.88	31.28 ± 5.52	0.053
TT (s)	14.68 ± 2.35	15.99 ± 16.49	0.184
D-dimer (mg/L)	0.3 ± 0.33	0.32 ± 0.27	0.720
FDP (mg/L)	0.91 ± 0.79	0.87 ± 0.59	0.704
IgA (g/L)	12.64 ± 2.51	10.22 ± 4.51	<0.001
IgG (g/L)	2.27 ± 0.8	3.94 ± 3.81	<0.001
IgM (g/L)	1.51 ± 0.62	1.48 ± 0.61	0.689
Complement 3 (g/L)	1.28 ± 0.22	1.39 ± 0.21	<0.001
Complement 4 (g/L)	0.34 ± 0.37	0.36 ± 0.1	0.683
ANA (+)	39 (12.9)	16 (20.8)	0.080
aCL IgG (+)	80 (26.5)	12 (15.6)	0.046
aCL IgM (+)	81 (26.8)	16 (20.8)	0.278
aβ2GP1 IgG (+)	78 (25.6)	16 (20.8)	0.360
LA1/LA2 ratio	1.09 ± 0.17	1.06 ± 0.13	0.158
Hcy (μmol/L)	12.08 ± 9.35	10.12 ± 6.01	0.082
25-hydroxyvitamin D (ng/mL)	12.47 ± 6.13	15.95 ± 7.46	<0.001

Variables are given as mean ± SD or frequency with percentages. (+) represents “positive”

*BMI* body mass index, *FBG* fasting blood glucose, *2hPG* 2-hours plasma glucose in the 75 g oral glucose tolerance test, *TC* total cholesterol, *TG* total glycerides, *HDL-C* high-density lipoprotein cholesterol, *LDL-C* low-density lipoprotein cholesterol, *TSH* thyroid-stimulating hormone, *FT-3* free triiodothyronine, *FT-4* free thyroxine, *TG-Ab* thyroglobulin antibody, *TPO-Ab* thyroid peroxidase antibody, *PT* prothrombin time, *APTT* activated partial thrombin time, *TT* thrombin time, *FDP* fibrinogen degradation products, *ANA* antinuclear antibody, *aCL* anticardiolipin antibody, *aβ2GP1* anti-β2 glycoprotein 1 antibody, *LA1* lupus anticoagulant (LA) screening test, *LA2* LA confirmatory test, *Hcy* homocysteine

### Logistic regression analyses revealed eight variables associated with pregnancy loss risk

Overall, we included 37 potential risk factors of subsequent pregnancy loss (Table 1), and the correlation heat map (Fig. S1) showed some correlation between some of these variables. Subsequently, the backward multivariable logistic regression model was used to screen the final predictors from these 37 variables. Given the statistic performance, eight variables were finally cooperated in the predictive model, per the following results: maternal age (odds ratio [OR] = 1.068, 95% confidence interval [CI]: 1.002–1.139), previous pregnancy losses (2 times: OR = 1.204, 95%CI: 0.642–2.260; 3 times: OR = 2.804, 95%CI: 1.213–6.486; ≥4 times: OR = 2.814, 95%CI: 1.008–7.852), TC (OR = 0.767, 95%CI: 0.571–1.030), aCL IgG (OR = 6.149, 95%CI: 0.668–61.654), aCL IgM (OR = 0.049, 95%CI: 0.005–0.505), C4 (OR = 3.118, 95%CI: 0.634–15.335), TPO-Ab (OR = 1.761, 95%CI: 0.904–3.428) and FT-4 (OR = 1.026, 95%CI: 0.964–1.093) (Table 2).

**Table 2:** Multivariable logistic model of probability of pregnancy loss for patients

Variables	OR (95%CI)
Maternal age	1.068 (1.002–1.139)
Previous pregnancy losses = 1	Ref.
2	1.204 (0.642–2.260)
3	2.804 (1.213–6.486)
≥4	2.814 (1.008–7.852)
TC	0.767 (0.571–1.030)
FT-4	1.026 (0.964–1.093)
TPO-Ab	1.761 (0.904–3.428)
aCL IgG	6.419 (0.668–61.654)
aCL IgM	0.049 (0.005–0.505)
C4	3.118 (0.634–15.335)

Variables are given as odd ratio (OR) with 95% confidence intervals (CIs)

*TC* total cholesterol, *FT-4* free thyroxine, *TPO-Ab* thyroid peroxidase antibody, *aCL* anticardiolipin antibody, *C4* complement 4

### Model development and validation

**Fig. 2:**
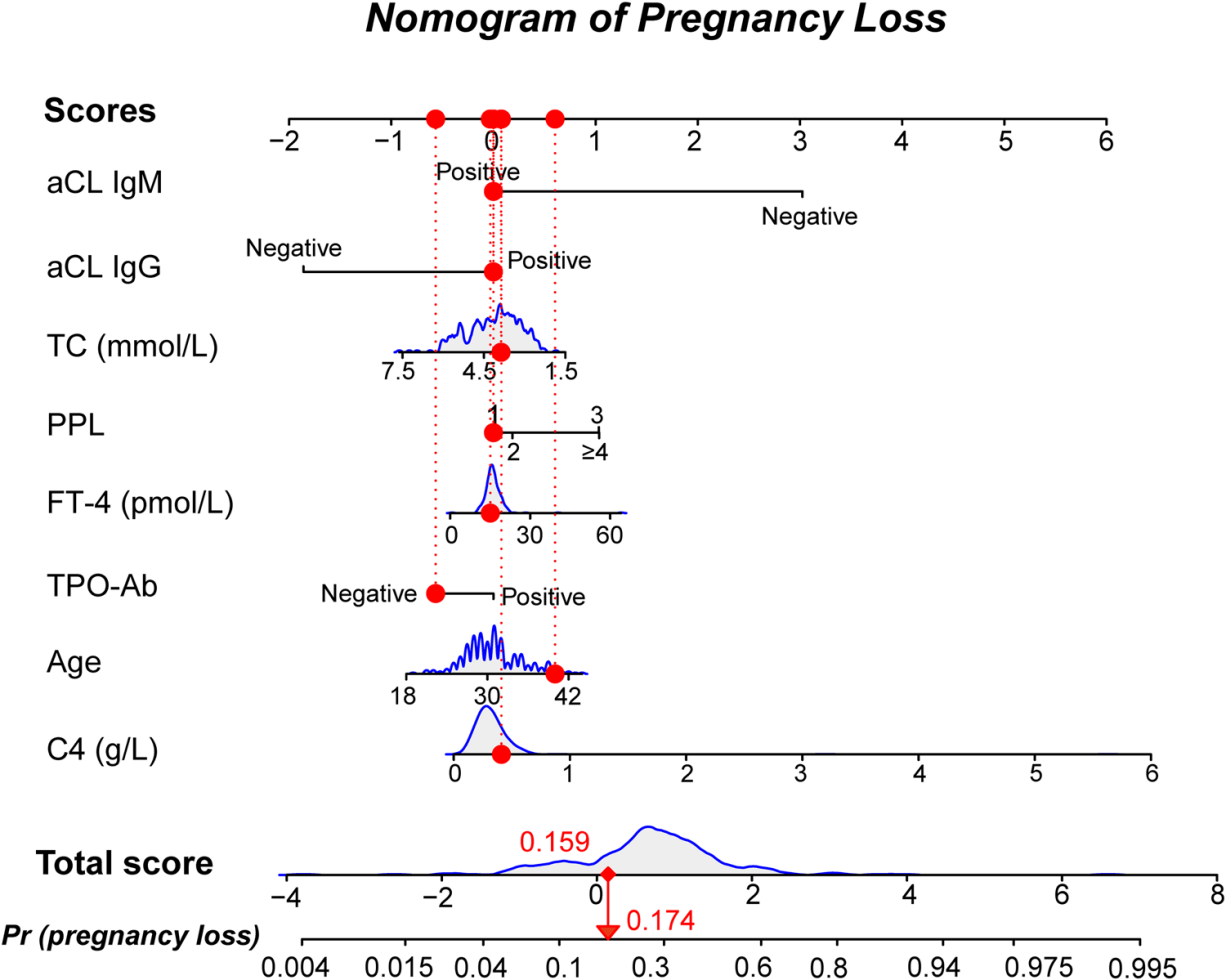
The predictive nomogram for pregnancy loss risk in women with a history of pregnancy loss. The eight factors are incorporated into the final predictive model, including maternal age, previous pregnancy losses (PPL), anticardiolipin antibody (aCL) IgG and aCL IgM, thyroid peroxidase antibody (TPO-Ab), total cholesterol (TC), free thyroxine (FT-4), and complement 4 (C4). Total scores are calculated by aligning the dots on each numbered row. For instance (expressed in red), the total score of this No.5 patient was 0.159, indicating that her pregnancy loss probability was 17.4%

**Fig. 3:**
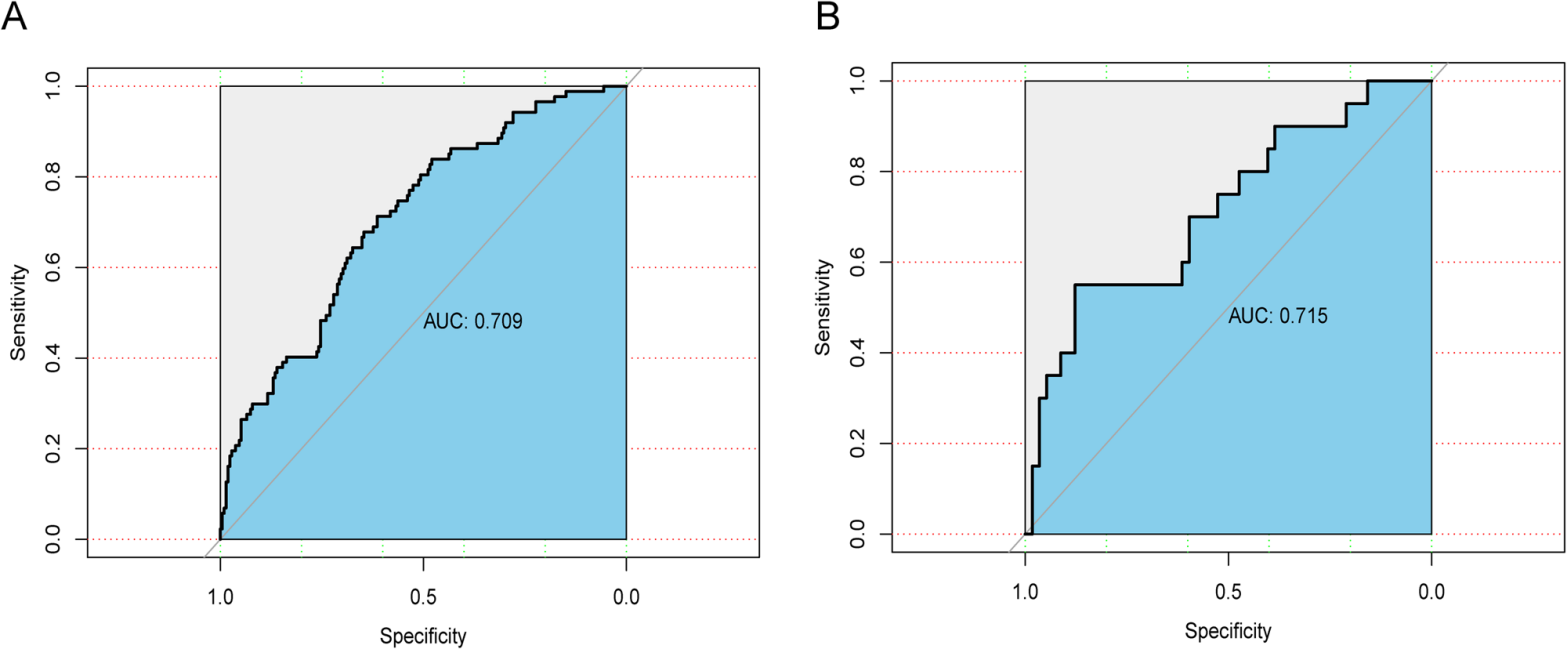
ROC curve and calibration curves of the nomogram. **A** ROC curve evaluating the discrimination of this prediction model for pregnancy loss probability in the training cohort, with 0.709 of the AUC value. **B** ROC curve evaluating the discrimination of this prediction model in the validation cohort, with 0.715 of the AUC value

**Fig. 4:**
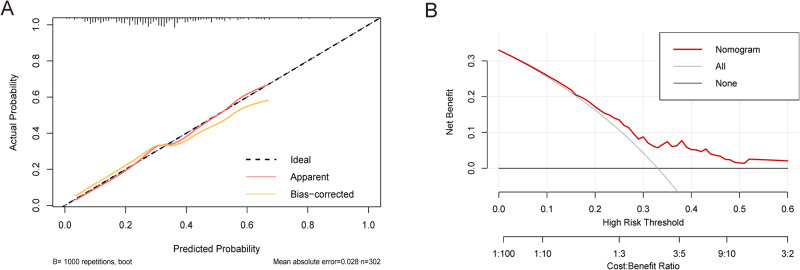
Clinical decision curve and clinical impact curves of the nomogram. **A** Calibration curve assessing the association between the predicted value and actual rates of pregnancy loss risk among these patients. **B** Clinical decision curve evaluating the net benefits of the predictive model for pregnancy loss risk in the patients with a history of pregnancy loss

A nomogram was developed for predicting pregnancy loss rates using the eight predictors (Fig. 2). For each patient, the higher the total scores, the higher the pregnancy loss risk. The area under the curve (AUC) of this predictive model was 0.709 (Fig. 3A). The internal validation of training cohort was performed by tenfold cross-validation repeated 50 times using bootstrap method, with an accuracy of 0.723 and a kappa value of 0.151. Also, the predictive model exhibited a well performance of discrimination in the validation cohort, with the AUC value of 0.715 (Fig. 3B). Calibration curve of the predictive model showed a high consistency between the actual values and predictive values, with a Chi-square value of 12.786 and a *P* value of 0.119 in the Hosmer–Lemeshow test (Fig. 4A). The clinical decision curve showed that the nomogram presented fair predictive capacity (Fig. 4B). Additionally, the clinical impact curve indicated that the nomogram could effectively classify the patient as positive (high risk) or true positive (high risk with the event) (Fig. S2). For example, when predicting 100 individuals at a risk threshold of 40%, the model would identify around 20 individuals as positive (at high risk of pregnancy loss), of whom about 15 would be true positives (at high risk and ultimately had pregnancy loss).

## Discussion

The nomogram developed in this study represents a novel approach to predicting the possibility of subsequent pregnancy loss for patients with a history of pregnancy loss and abnormal glucose/lipid metabolism. Furthermore, this is also the first predictive model constructed to estimate pregnancy loss risks for these patients. Our predictive nomogram incorporated eight pre-pregnancy parameters, including maternal age, previous pregnancy loss, TC, FT-4, TPO-Ab, aCL IgM, aCL IgG and C4. The ROC and calibration curves demonstrated that the nomogram has satisfactory discriminatory and calibration abilities.

It is well-known that maternal age and previous pregnancy loss are independent risk factors for pregnancy loss. In particular, the risk of subsequent pregnancy loss can reach over 80% after three or more pregnancy losses [9]. Our findings of multivariable logistic regression are consistent with previous studies that maternal age (OR = 1.068, 95%CI: 1.002–1.139) and previous pregnancy losses (3 times: OR = 2.804, 95%CI: 1.213–6.486; ≥4 times: OR = 2.814, 95%CI: 1.008–7.852) were the risk factors of pregnancy loss in these patients. The results of prospective studies of patients with RPL also revealed a high degree of consistency—the risk of subsequent pregnancy failure increased with advanced age and an increased number of previous pregnancy losses [15, 16].

Evidence indicates that maternal lipid metabolism disorders raise the risk of adverse pregnancy outcomes [17, 18]. Low cholesterol levels are generally considered healthy, while extremely low cholesterol levels have been linked to adverse pregnancy outcomes [19]. The animal studies conducted by Tozawa et al. indicated that prepregnancy cholesterol levels below the normal range may be related to an increased risk of neural tube defects, which can affect the development of the embryo’s brain or spinal cord [20]. Furthermore, cholesterol is a major component of cell membranes and performs critical functions in the processes of cell division and the formation and function of fetal organs [21, 22]. Changes in membrane function can significantly impact the transport of various compounds acquired by the fetus through membrane-mediated processes, including lipids, amino acids, and glucose [23]. Reduced cholesterol levels may hinder these processes, leading to inadequate fetal development and an increased risk of premature birth [24]. On the other hand, obesity may increase the risk of pregnancy loss [25]. The risk of pregnancy loss before the first-born child of overweight women has been reported to be as high as 25–37% [26]. Studies found that elevated levels of cholesterol or triglycerides before the third trimester of pregnancy may result in an increased risk of preterm delivery [27, 28]. Besides, pre-pregnancy overweight and obese women are significantly more prone to pregnancy complications such as gestational hypertension and gestational diabetes mellitus than normal-weight women, resulting in significantly lower live birth rates [29, 30].

Thyroid dysfunction is also associated with the occurrence of adverse pregnancy outcomes such as preterm delivery and pregnancy loss, and nearly 0.5% of pregnant women have combined hypothyroidism [31]. Decreased FT-4 levels are commonly employed to diagnose hypothyroidism [32]. Previous studies have shown that decreased thyroid function is related to RPL because severe hypothyroidism could lead to anovulation and infertility. Maternal hypothyroidism during pregnancy may also cause lower fetal birth weight and growth retardation [33]. Besides, a triple risk of pregnancy loss has also been reported in women with TPO-Ab [34]. About 28.8% of RPL patients test positive for thyroid autoantibodies, and the prevalence of subclinical hypothyroidism was significantly higher in RPL patients with TPO-Ab positive compared to those with TPO-Ab negative (52% vs. 16%) [35, 36]. It is therefore essential to maintain normal maternal thyroid hormone levels for normal fetal development. Current published consensus and guidelines emphasize the importance of screening for thyroid disease in patients with RPL. By assessing TPO-Ab and FT-4 levels, abnormal thyroid function can be timely detected, and normalizing thyroid function before conception may improve pregnancy outcomes for patients.

In addition, the presented study incorporated three immune-related predictive factors of aCL IgG, aCL IgM and C4. Antibodies are a part of the immune system and can react against their own tissues or embryonic tissues, triggering an immune-inflammatory response. This immune-inflammatory response poses a potential risk to normal pregnancy development by interfering with endometrial health and embryo implantation, thus increasing the likelihood of pregnancy loss. A meta-analysis study reported that aCL is a significant risk factor for pregnancy loss. Additionally, aCL was found in 5–51% of patients who experienced RPL [37, 38]. Positive aCL has been linked to the possibility of placental immunoinflammation, which can ultimately impact pregnancy outcome [39]. Consistent with previous research, our study findings indicated that aCL IgG and aCL IgM are risk factors for pregnancy loss [37, 40]. Wharfe et al. reported a prevalence of 38.4% for positive aCL IgG tests among patients with spontaneous abortion [41]. In a group of healthy pregnant women, Lynch et al. found a significant association between aCL IgG and pregnancy loss after adjustment for confounders [42]. Furthermore, Cronin et al. demonstrated a strong correlation between IgM aCL and RPL and noted that significant associations exist between high levels of IgG and IgM aCL and fetal loss [43]. However, the regression analysis conducted in this study revealed that aCL IgM acted as a protective factor against the risk of pregnancy loss (OR = 0.049, 95% CI: 0.005–0.505). A study conducted by Nielsen et al. found that aCL IgM positivity had a stronger association with pregnancy outcomes in RPL patients, and it had a significant impact on live birth rates (OR = 0.34, 95% CI: 0.2–0.7) [44]. Overall, our findings suggested that aCL IgM may increase the risk of pregnancy loss; although the risk of pregnancy loss is relatively lower in aCL IgM positive patients compared to aCL IgM negative patients (Fig. 2). Further research is necessary to thoroughly investigate the possible association between aCL IgM and the risk of pregnancy loss in these patients.

On the other hand, the complement system is also a part of the immune system, and it plays critical roles in the immune response, including the formation of the membrane attack complex and the release of inflammatory mediators [45, 46]. Pregnancy loss is linked to complement activation among some patients with or without RPL [47]. The C4 protein levels were significantly increased in women with RPL compared to the women without RPL [48]. Extraembryonic tissues, particularly the trophoblast, serve as the interface for maternal complement injury risk during pregnancy [49]. C4 is a particularly important component of the complement system that contributes to the formation of C3 convertase, which is pivotal to the activation of the complement system [50]. Immune-inflammatory response could cause an increase in C4 levels as a reaction to inflammatory signals within the body. Studies have shown that C4 protein levels are significantly higher in RPL patients compared to non-RPL women [48]. Besides, Ogasawa et al. found that women with higher C4 levels, especially those who had experienced pregnancy loss, had a diminished probability of achieving a successful pregnancy with a live birth [51]. They found that ~21% of patients (45/215) ultimately experienced pregnancy loss and had significantly elevated levels of C4, suggesting that C4 levels potentially could serve as a predictor of subsequent pregnancy outcomes in these patients.

This study has several strengths. To our best knowledge, this is the first study to develop a predictive nomogram model for evaluating the risks of pregnancy loss in women with abnormal glucose/lipid metabolism who also had a history of pregnancy loss using pre-pregnancy endocrine factors. Better news was that the predictive model presented a satisfactory performance. Couples who are ready for their next pregnancy, especially those who have previously experienced RPL, have the potential to benefit from this predictive model. All predictors included in the model are pre-pregnancy parameters that are routinely tested, with readily accessible data. Besides, using pre-pregnancy indicators to predict pregnancy outcomes is a prospective approach that enables timely patient management. If patients get high scores by the model, it may prompt clinicians to increase close attention to their pregnancy progress and promptly address potential problems. Furthermore, the application of this predictive model could optimize the allocation of medical resources, allowing high-risk patients to receive higher-quality care and support in their subsequent pregnancies, thereby enhancing the efficiency and effectiveness of pregnancy management.

However, our study has some limitations. Firstly, this is a retrospective study, which may be prone to recall bias due to its inherent limitations, despite our comprehensive follow-up analyses for all participants. Secondly, due to global variations in BMI calculations, our developed predictive model is currently better suited for Asian populations, especially the Chinese population, given the obesity criterion set in this study requires a BMI ≥ 28 kg/m^2^ [10]. Thirdly, our external validation was not very rigorous. However, the patients of the validation cohort were enrolled from the same hospital but were admitted at different periods, and their baseline and clinical characteristics were different. Still, the AUC value of the validation cohort was greater than 0.7, suggesting that the predictive model shows well extrapolation. In our future work, we will consider utilizing multi-center data and/or incorporating diverse ethnicities to further refine and enhance the applicability of the predictive model.

## Conclusions

In the current study, a nomogram was constructed for the first time to predict the risk of subsequent pregnancy loss in patients with a history of pregnancy loss and abnormal glucose/lipid metabolism based on the pre-pregnancy endocrine and immunological factors (maternal age, previous pregnancy losses, TC, FT-4, TPO-Ab, aCL IgG, aCL IgM and C4). The predictive model exhibits a favorable predictive performance and can assist clinicians in personalizing the management of subsequent pregnancies in these patients.

## Supplementary information

Supplementary materials

## Data Availability

The datasets used and/or analyzed during the current study are available from the corresponding author on reasonable request.
