# Supplementary material for: A predictive model of pregnancy loss using pre-pregnancy endocrine and immunological parameters in women with abnormal glucose/lipid metabolism and previous pregnancy loss
Source: Endocrine. 2024 Jun 19;86(1):441–50. doi: 10.1007/s12020-024-03937-7 (PMC11445311; doi:10.1007/s12020-024-03937-7)
Supplement: Supplementary file 1 — Supplementary materials [file 12020_2024_3937_MOESM1_ESM.docx]

**Supplementary materials**

**Fig S1.** Heatmap of correlations among the 42 candidate predictors


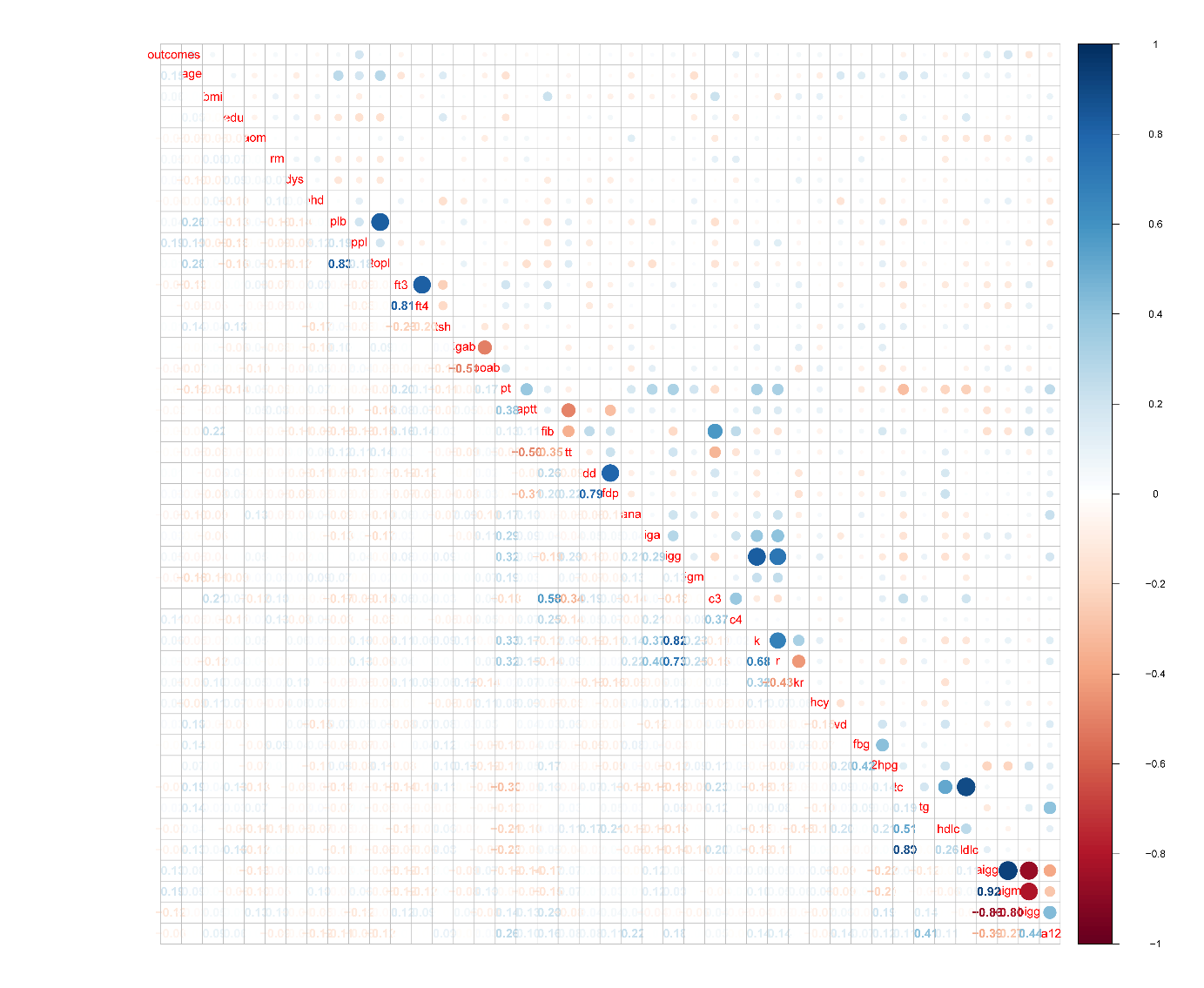


The color is more pronounced if there is a strong correlation between the variables. Red indicates a negative correlation, while blue indicates a positive correlation among the variables.

**Fig S2.** Clinical impact curve of the predictive model.

**
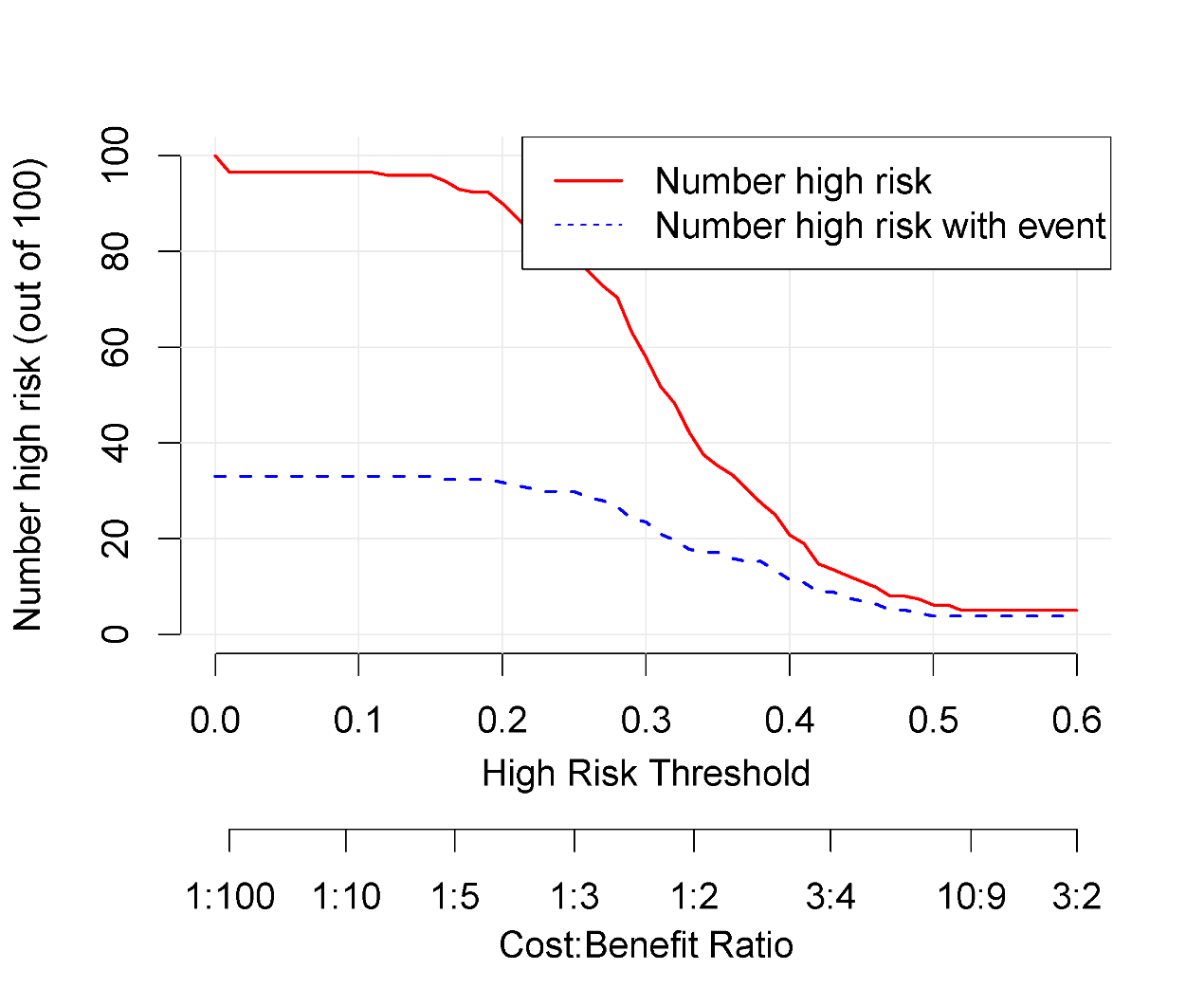
**

Clinical impact curve showing the number of patients classified as positive (high risk; red line) and true positive (risk with event; blue line) by the prediction model.
